# Night Terrors and Nightmares

**Published:** 1899-08-26

**Authors:** 


### NIGHT TERRORS AND NIGHTMARES.

Writing on the subject of night terrors in children Dr. Graham Little[1] points out that his colleague, Dr. Coutts,[2] of the East London Children's Hospital, has drawn an interesting distinction between night terrors and nightmares. In one class the disturbance of sleep is due to reflex stimulation of the sleeping child, in the other the restlessness is caused by disordered central impulses; the first may be included among nightmares; the second, that is those arising from central impulses, constitutes true night terrors. This classification would correspond pretty closely with that suggested by Silbermann,[3] who, dividing such cases into two classes, placed in the first class those which are secondary to some other disorder, mostly of digestion, while in the second class he placed those cases to which no such causes could be ascribed. These he called idiopathic, and assumed to be due to disordered brain functions in a neurotic child. But, in fact, many people have worked at this subject, and while it has been generally recognised that in a large number of cases such disturbances of sleep are associated with various fairly obvious excentric causes, most observers have found a residuum of cases in which, to say the least, such causes have not been evident. Dr. Graham Little has then set himself to inquire whether these "terrors" are really centric, and really depend on some primary nerve defect, such as may be associated with epilepsy, as has been thought by some to be the case, or whether in the night terrors, as in the nightmares, some outside cause may not be in operation. The results of his investigation are interesting. He argues that night terrors are caused by a reflex stimulation produced by dyspnœa, moderate in degree but prolonged, occurring in the early hours of sleep of young children. The stimulus is usually of the character of what Bain called an organic sensation, vague in its mental character as compared with the stimulations of special sense, but attended by voluminous though ill-defined feeling, which, "in the child's fantastic turmoil of subconsciousness," is translated into terror, and is associated with an external cause. The hallucinations thus produced are mostly visual, but not always, A preponderating number of these cases of night terrors are found in rheumatic subjects with early heart disease, and a considerable proportion of the cases are due to obstruction in the nasal cavities and fauces. Digestive disorders, however, do not play the important part which is often assigned to them in their causation, and the evidence for the connection of such cases with epilepsy or allied neurosis is scanty.

[1] Brit. Med. Journ., Aug. 19. [2] Amer. Journ. of Med. Sci., Feb., 1896. [3] Jahrbuch für Kinderheilkunde, 1893.

